# A cross-lingual analysis on the spread of misinformation using the case of Ivermectin as a treatment for Covid-19

**DOI:** 10.1038/s41598-023-41760-8

**Published:** 2023-09-06

**Authors:** Cameron Lai, Fujio Toriumi, Mitsuo Yoshida

**Affiliations:** 1 Graduate School of Engineering, The University of Tokyo, Tokyo, 113-0033 Japan; 2 Institute of Business Sciences, University of Tsukuba, Tokyo, 112-0012 Japan

**Keywords:** Computational science, Information technology, Epidemiology

## Abstract

The spread of misinformation transgresses international boundaries, between languages and cultures. This is especially evident in times of global crises such as the Covid-19 pandemic. This study observes misinformation on Twitter in the Japanese and English languages regarding false claims that the drug Ivermectin is an effective treatment for Covid-19. Our exploratory cross-lingual analysis identifies key themes of discussion and influential users in both languages, finding English misinformation to be highly popular amongst Japanese users. Significantly, an analysis of the timing of retweets between languages reveals that Japanese users find and widely share English misinformation often before English users themselves. This contradicts expectations that users from other languages tend to pick up on popular misinformation in English. Instead, they seek out English language sources irrespective of their popularity to support their agenda. These results emphasise the importance of cross-lingual mitigation strategies for organizations trying to combat misinformation, and that they must look beyond their own language spheres.

## Introduction

The overwhelming spread of the Covid-19 (SARS-CoV-2) virus has resulted in millions of deaths around the world. Exacerbated by factors such as vaccine distrust and availability issues, users on social media have been advocating for alternative treatments, often with no proven effectiveness^1–4^.

One such treatment is the drug Ivermectin. Initially conceived as an anti-parasitic agent to treat livestock, it has found widespread use in humans as a treatment for river-blindness^5^. The interest in Ivermectin as a treatment for Covid-19 was sparked by the work done by ref^6^ in 2020, where they find Ivermectin to be an effective inhibitor of the virus in vitro. Studies have since tried to prove a link between Ivermectin and a reduction in Covid-19 mortality; however none have been able to convincingly show this in Randomised Controlled Trials (RCTs), which are considered the “gold standard” for verifying the efficacy of medical interventions^7^. There has been intense debate, and studies that claim effectiveness have often been criticised as being of low quality, and in some cases fraudulent, with a number of papers having been retracted^8–10^. At the time of writing, the largest RCT to date, the Together Trial, has found no evidence of the effectiveness of the drug to treat Covid-19^11^. The World Health Organization (WHO) and American Food & Drug Adminisatration (FDA) also continue to discourage consumption to treat Covid-19^12,13^.

**Table 1:** Sample of top keywords demonstrating the popularity of alternative treatments in both languages for September 2020. Full tables for data collection period are represented in Tables 4 and 5.

English			Japanese		
Rank	Keyword	Count	Keyword	English translation	Count
1	Zinc	368		Japan	1,012
2	Study	325		Treatment	758
3	Borody	263		Avigan	586
4	Says	229		Side effects	548
5	Thomas	220		Medicine	536
6	Professor	211		Overseas	494
7	Doxycycline	202		Symptoms	466
8	Gastroenterologist	200		This	454
9	Hydroxychloroquine	197		Doxycycline	440
10	Treatment	193		Kitasato University	418

Despite these warnings, Ivermectin continues to be suggested as a treatment for COVID-19 in social media. The discourse generated in both academic and non-academic circles means that it is a highly complex piece of misinformation, and also with significant public health ramifications. This work sits amongst others that have investigated specific instances of misinformation in regards to Covid-19^14,15^, and in the wider public health sphere itself such as those done in regards to the Zika virus^16^ and the HPV (Human PapillomaVirus) vaccine^17^.

In addition to English, significant discourse also takes place in the Japanese language. Other research has characterised Covid-19 misinformation across languages, finding that health-based misinformation to be one of the key themes that appear in multiple languages^18^. It therefore comes as no surprise that health misinformation is also prevalent in Japanese. The popularity of Ivermectin in Japanese may be explained by the fact that the discoverer of the drug was Japanese. This setting provides a unique opportunity to analyse how misinformation spreads between the English and Japanese social media communities. We therefore analysed misinformation related to “Ivermectin as a treatment for Covid-19” in English and Japanese. We compared emergent themes, influential users, and analysed how misinformation is shared between the two languages. Our results showed that English language misinformation is popular amongst Japanese users, and is shared independently of trends within English users. In doing so, our research contributes to a deeper, more detailed understanding of how misinformation is shared in a cross-lingual setting.

**Table 2:** Sample of top node degree English users from July to September 2021. Demonstrates an increase in anti-use users in August 2021. Full information for data collection period are represented in Tables 6 and 7.

Rank	July 2021			August 2021			September 2021		
	User	Degrees	Stance	User	Degrees	Stance	User	Degrees	Stance
1	Individual	4501	Pro-use	Journalist	8342	Anti-use	Journalist	13,294	Anti-use
2	Doctor	3883	Pro-use	Doctor	8012	Pro-use	doctor	8255	Pro-use
3	Individual	3701	Pro-use	Individual	6106	Anti-use	Individual	7084	Pro-use
4	Group	2,693	Pro-use	Individual	5092	Anti-use	Individual	6823	Undetermined
5	Individual	2377	Pro-use	Individual	4697	Pro-use	Doctor	5424	Pro-use
6	Individual	2096	Pro-use	Group	4603	Pro-use	Doctor	5345	Pro-use
7	Group	1751	Pro-use	Individual	4262	Anti-use	Individual	5291	Anti-use
8	Individual	1578	Anti-use	Individual	4096	Pro-use	Group	5055	Anti-use
9	Individual	1387	Pro-use	Individual	4096	Pro-use	Doctor	4863	Anti-use
10	Individual	1371	Undetermined	Individual	3074	Anti-use	Doctor	4509	Pro-use

**Table 3:** Example of a URL containing misinformation that has been properly translated by pro-use users in two separate retweets.

URL	URL content summary	Translated retweet text content
https://www.firstpost.com/health/ bangladesh-medical-team-says-ivermectin-with-antibiotic-doxycycline-works-to-treat-covid-19-patients-8381321.html	Doctors in Bangladesh claim that a combination of Ivermectin and Doxycycline helps to treat Covid-19	Report that ivermectin was surprisingly effective against the new coronavirus in Bangladesh. Immediately after being found positive, it was administered together with the antibiotic doxycycline, and the patient recovered within 4 days and became negative.
		Bangladesh. A combination of ivermectin and the antibiotic doxycycline is effective in treating corona patients. He has tested 60 patients and reported that all have recovered. The patient tested negative in 4 days and had a 50% reduction in symptoms in 3 days. There are no side effects. Currently, it is proposed as a therapeutic drug.

## Results

Our results are summarised into three main sections. The first is a content analysis, where we observe the general themes related to Ivermectin that appear during the course of the pandemic, and draw connections to the events that occurred in the English and Japanese worlds (Table 1). The next section identifies influential users and the role that they played in the Ivermectin discourse. This includes whether they were Ivermectin pro-use or anti-use, affiliated groups, and their influentiality (Tables 2 and 3). Finally, we observe how Japanese and English Ivermectin pro-use users (misinformation spreaders), share misinformation across the two languages. We do so by using the language of URLs that were retweeted. We compare the language and popularity of retweeted URLs in relation to the native language of the user, and the timing of when URLs were shared by non-native users compared to native users.

### Content analysis

Performing a straightforward keyword count on collected retweets shows that much of the content reflects an agenda that supports the use of Ivermectin to treat Covid-19. The global nature of the pandemic means that it is of little surprise that similar themes occur in both English and Japanese, despite the obvious cultural differences (Tables 4 and 5). For example, alternative treatment methods are mentioned often, with hydroxychloroquine mentioned in both languages. Within each language, doxycycline and zinc are popular in English, and Avigan, an antiviral medicine used to treat influenza, in Japanese (Table 1). Users from both languages also picked up on how Ivermectin was being used overseas, particularly as it became a part of official treatment guidelines in some countries. This was the case in the Indian state of Goa, and Brazil amongst other South American countries^19^.

**Table 4:**
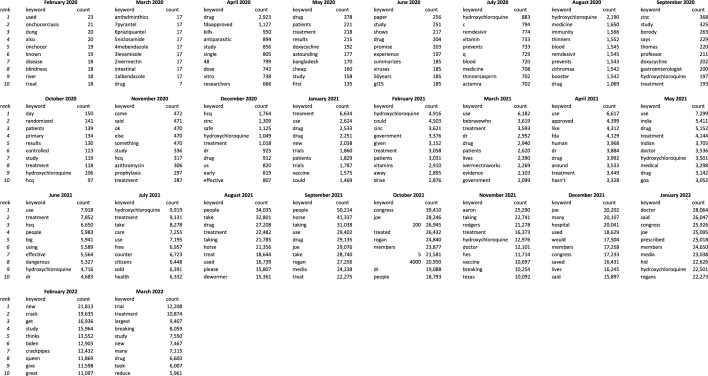
Top 10 keywords by month in English.

**Table 5:**
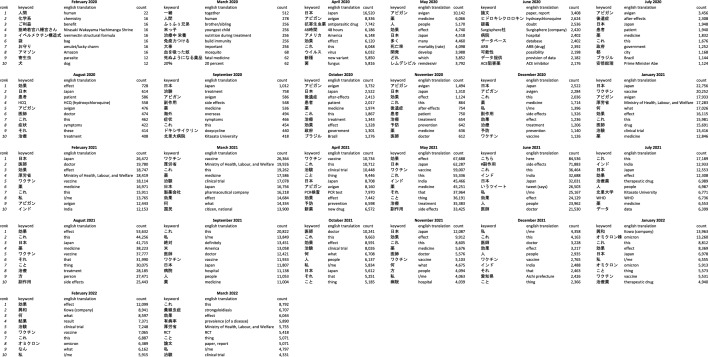
Top 10 keywords by month in Japanese.

A high level of criticism is also levelled at government institutions, reflecting a low level of trust. English users are critical of agencies such as the FDA, and the American Congress. In March 2021, the hashtag “bebravewho” also became popular, which was a call on the World Health Organization to list Ivermectin as an approved drug for treating Covid-19 in the face of perceived hesitancy. Japanese users on the other hand, mention the Ministry of Health, Labour, and Welfare, the Japanese government agency responsible for the administration of health amongst other areas. They express frustration at the lack of perceived action from the agency in making Ivermectin an approved drug to treat Covid-19.

Exclusive themes also exist within each language that reflect the current affairs regarding Ivermectin at the time. For English users, the word “horse” appears around August 2021, reflecting news at the time that people were falling ill due to taking Ivermectin used for animals instead. There are also complaints amongst Ivermectin advocates that the media branded Ivermectin as strictly not for human use, despite the drug also being available in variations for human consumption. The keywords “Joe” and “Rogan” also appear, referring to a commentator known for spreading Covid-19 misinformation, and who took Ivermectin after contracting Covid-19. Japanese content tends to be more medically oriented. Some of the specific events that arose during the pandemic also become apparent. For example, “Kitasato” and “Kowa” are references to Kitasato University and the Kowa pharmaceutical company. During the pandemic, Kitasato University conducted joint research with Kowa into the viability of Ivermectin. The results were heavily anticipated by Ivermectin supporters in Japan ; however, no proven efficacy was found in their research^20^.

### Influential users

Reflecting the content analysis, discourse is dominated by users who champion the use of Ivermectin as a treatment for Covid-19 over the course of the pandemic in both English and Japanese. It is not until around August 2021 that users who are against the use of Ivermectin, or anti-use, users become more influential (Tables 2, 6 and 7).

**Table 6:**
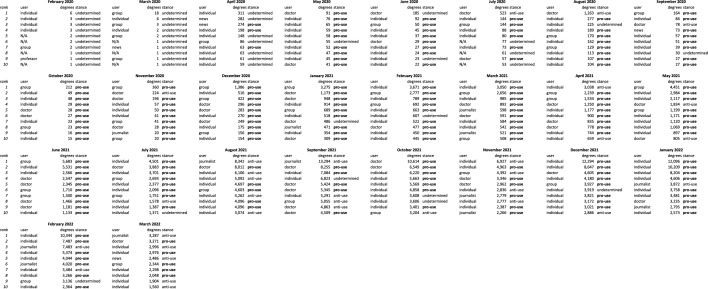
Top 10 node degree users by month in English.

**Table 7:**
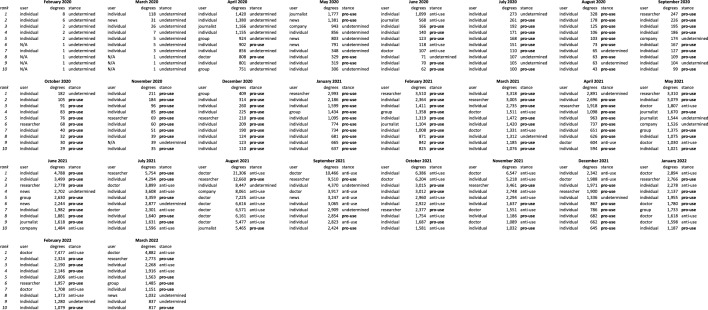
Top 10 node degree users by month in Japanese.

**Figure 1:**
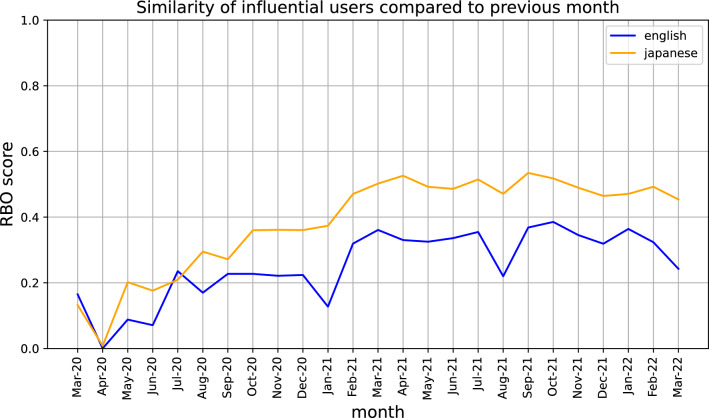
RBO similarity score of users between each month. Users are ranked by influentiality, therefore a higher RBO score on the y-axis indicates fewer changes in top influential users month to month. Increasing RBO over time demonstrates development of core influential user group in each language as pandemic progresses.

**Figure 2:**
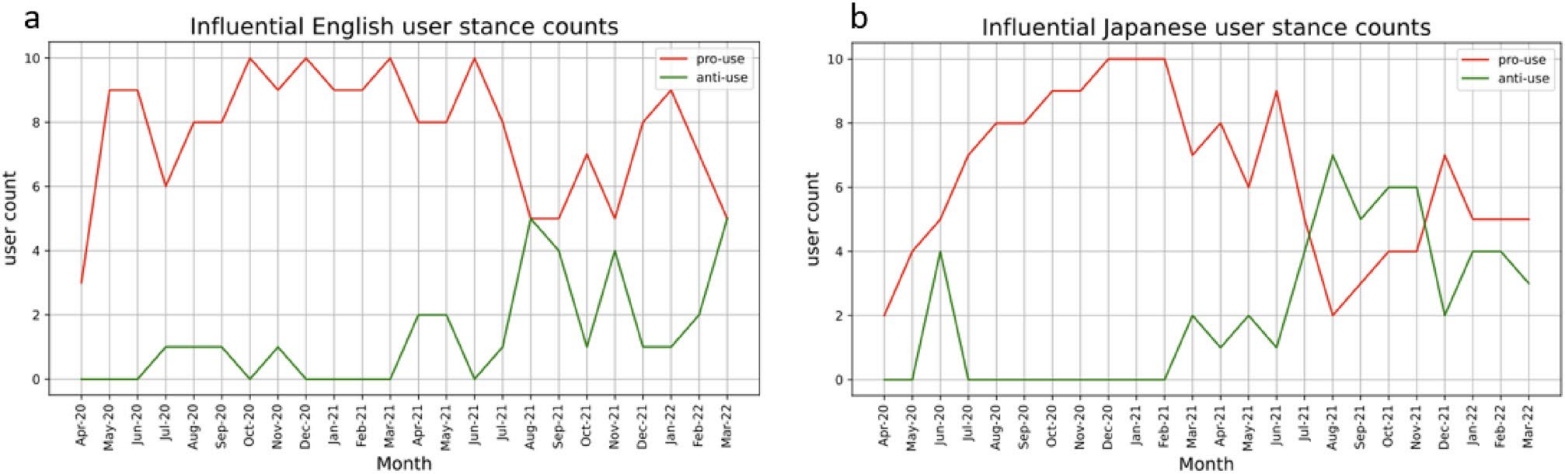
Number of Ivermectin pro-use and anti-use (stance) of top 10 influential users each month amongst (**a**) English users, and (**b**) Japanese users. Both graphs show dominance of influential pro-use users (red line) until a spike in anti-use users (green line) in August 2021, though it quickly decreases again.

To quantify how influential users change between months, we made use of Rank Biased Overlap (RBO), which is a method to calculate the similarity of two ranked lists, shown in Fig. 1. A higher RBO score on the y-axis indicates greater similarities between the influential users from the previous month and the current month (x-axis). The steadily increasing trend lines for both languages, combined with the fact that Ivermectin pro-use users are the most influential, demonstrate that prominent pro-use users develop and maintain their influentiality throughout the pandemic. This increasing trend continues for users from both languages until around August 2021, when there is a sudden increase in appearances of Ivermectin anti-use users (Fig. 2, Table 1). It is quickly reversed, with the core group of pro-use users in each language remaining dominant until the end of the observation period (Tables 6 and 7).

The higher Japanese trend line in Fig. 1 suggests that more of the same influential users in Japanese appear month to month compared to their English counterparts. This would suggest that there is a more coherent community of misinformation spreaders in Japanese. However, we cannot discount the impact that Twitter account suspensions may have had in removing certain users. This would result in a less coherent English community. During our investigations into verifying the owners of the accounts, we noted that a number of accounts had been suspended for violating Twitter’s Covid-19 misinformation guidelines, although many accounts have since been reinstated at the time of writing. Concerningly, a number of the top Ivermectin pro-use users in English were also identified as medical doctors (Table 6). Many of these accounts could be linked or made reference to the Frontline Covid Care Alliance (FLCCC), an organization notorious for spreading Covid-19 misinformation, with one individual having taken part in the storming of the Capitol on 6 January 2021^10^. Despite these associations, the low coherence could be explained by the aforementioned account suspensions, as well as the high variation of other users that include medical professionals not linked to the FLCCC, prominent regular users, politicians, and journalists. Despite the greater coherence in pro-use Japanese users, their associations with each other appear to be more fractured. There was no indication that any of the Japanese users were affiliated with with each other through a higher organization, and a large number go through efforts to anonymize themselves. On the other hand, Japanese anti-use users are more transparent, with the majority being medical professionals.

### Cross-lingual misinformation

In order to observe how misinformation spreads within the English-Japanese cross lingual context, we identified pro-use users in the wider retweet network based on their interactions with the tagged influential users in the user influentiality analysis. Of the 698,484 unique users in English and 259,151 in Japanese, 57 percent of English users were tagged as pro-use, and 55 percent in Japanese. We then extracted the URLs that were shared by both English and Japanese users, identified the language of the URLs, and compared the URL retweet popularity, influentiality of users who shared URLs in their non-native language, and the timing of when URLs are shared between languages.

**Figure 3:**
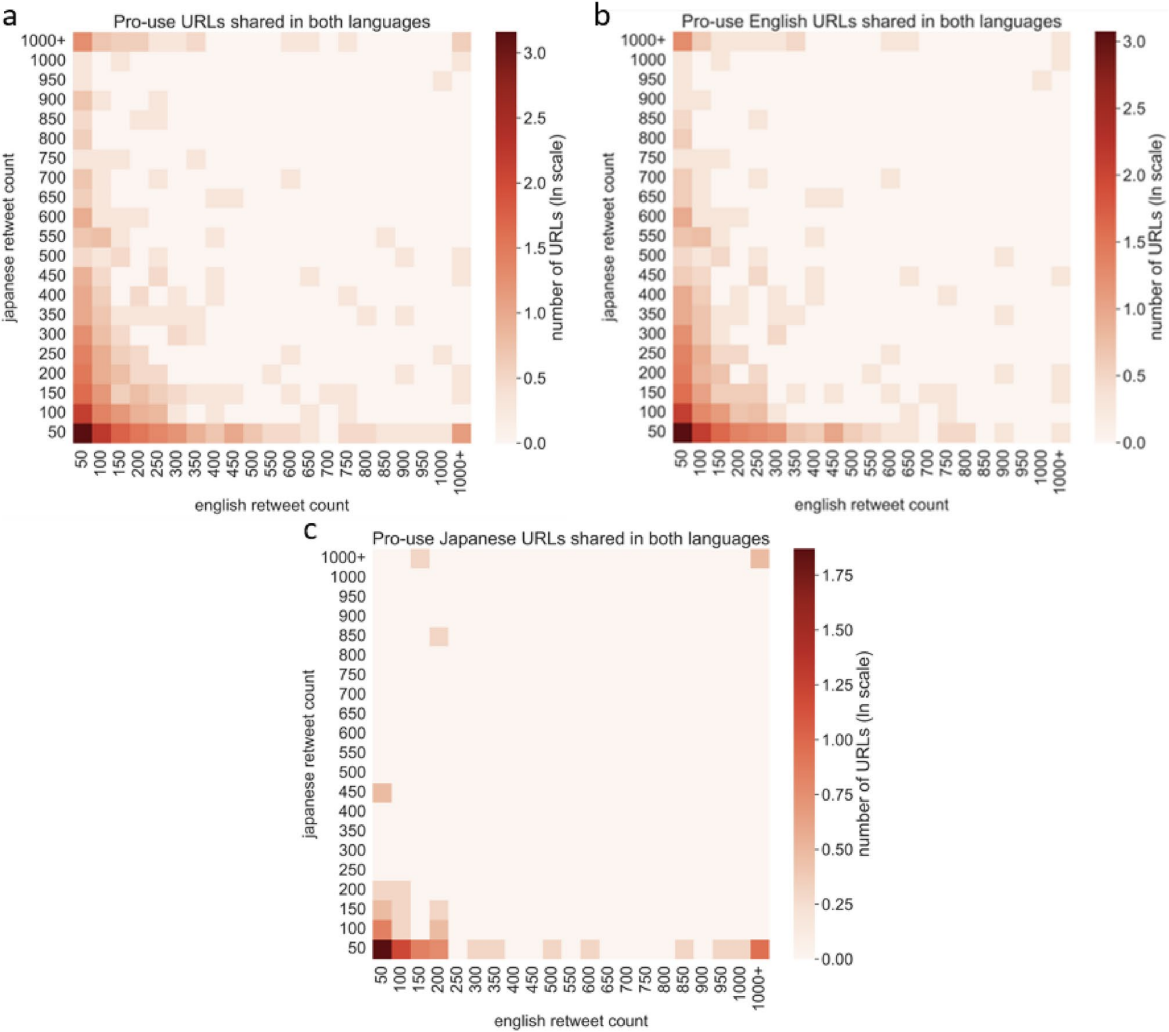
Retweet count of URLs shared by both Japanese and English users. (**a**) All URLs shared by both users, (**b**) English language URLs shared by both users, (**c**) Japanese language URLs shared by both users.

**Figure 4:**
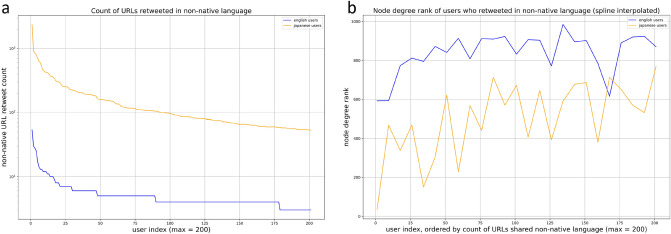
(**a**) Top 200 users ordered by count of URLs shared in non-native language, and (**b**) node degree of the top 200 users identified in (**a**).

The majority of URLs are shared a low number of times in both languages, which is demonstrated by the dense number of URLs in the bottom left of each graph in Fig. 3. In other words, the majority of URLs had low retweet counts amongst both English users (x-axis) and Japanese users (y-axis). Most of the URLs shared by both English and Japanese users were English, hence the visual similarities between subgraphs 3a and b. The density of the Japanese user retweet counts along the y-axis of Fig. 3b also shows that English misinformation is popular amongst Japanese users. On the other hand, Japanese language URLs that have been retweeted in both languages are not as popular amongst either the Japanese or English communities, suggesting that English users do not necessarily share URLs that are popular amongst Japanese users. Further, the lack of Japanese URLs amongst English users can be explained by the low volume of non-native URLs shared by English users, and the low level of influentiality of English users who share non-native URLs. In analysing the users who share the most URLs in their non-native language, Fig. 4a shows that Japanese users share significantly higher volumes of non-native URLs than English users . Further, Fig. 4b shows that those same users are also much more influential (lower node degree ranking) than their English counterparts. The much lower volumes of non-native URLs shared by English users and their relatively low levels of influentiality help explain the non-existent popularity of Japanese URLs amongst English users.

Throughout the investigation of URLs posted by non-native pro-use users, we found little evidence to suggest language mis-translation in order to support a misinformation agenda. URLs tended to be faithfully translated, with Table 3 being one such example. This could be explained by the fact that the content of information posted in another language can be easily checked using translation service tools. If users are found to be purposefully mis-translating content, they could quickly lose their credibility. Further, the abundance of misinformation supporting the use of Ivermectin suggests that there is little incentive to manipulate content that does not support the agenda. Overall, these results confirm the popularity of English misinformation amongst the Japanese Twitter community.

**Figure 5:**
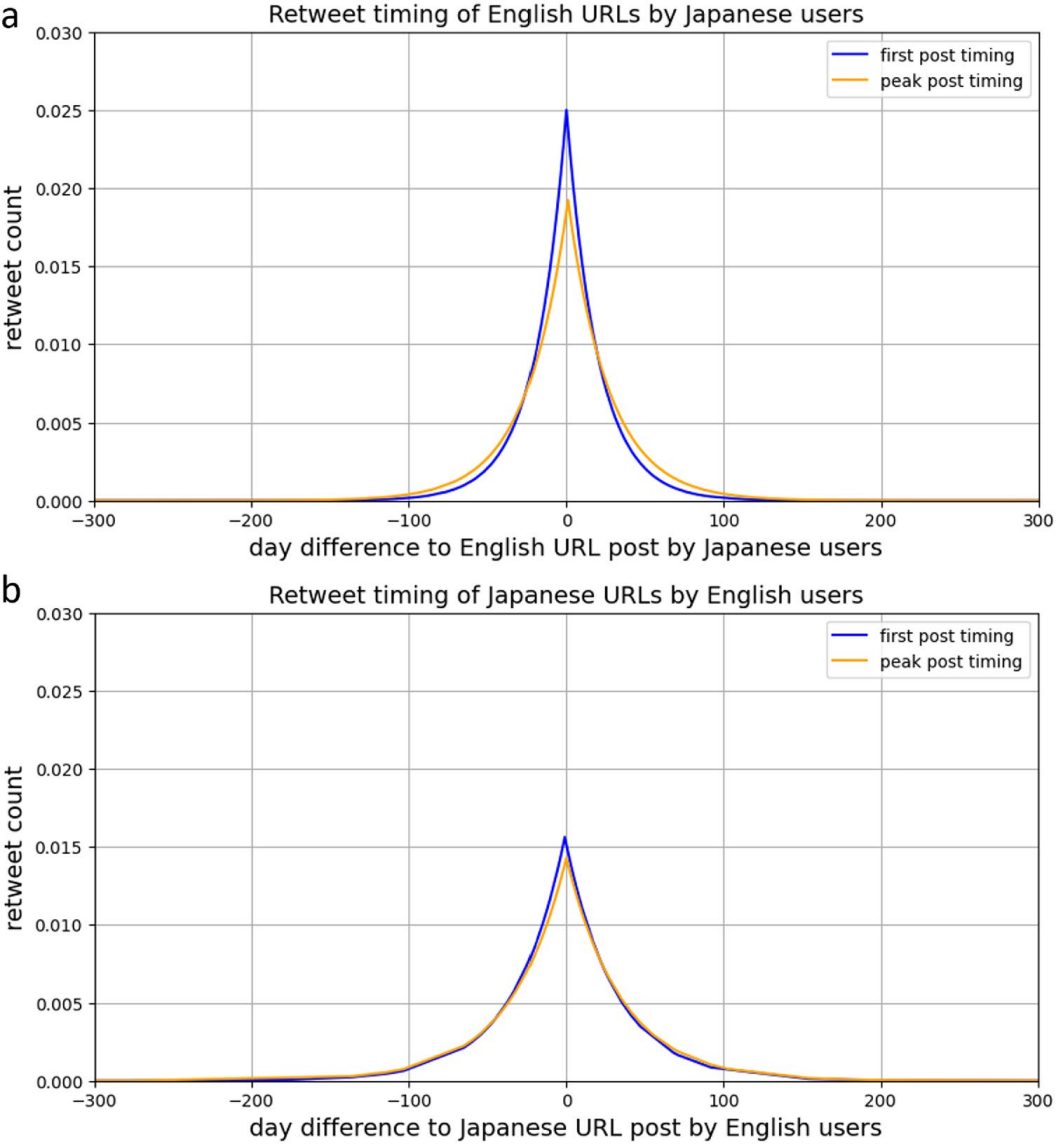
Distribution of retweet timing of URLs by non-native language users relative to timing by native language users. Negative day differences indicate URL was first posted and/or reached peak daily retweets in non-native language prior to being posted in native language.

Finally, we find that a significant number of English language URLs are posted and spread by Japanese pro-use users before English users. Figure 5 shows the retweet timing distribution of when URLs in one language are first posted (blue line), and reach peak retweet diffusion (orange line), i.e. maximum daily retweet count in the other language by non-native users, relative to the time it is first posted by native users. In the example of an English language URL being posted by an English user at day 0, the blue line represents when the URL was first posted by a Japanese user, and the orange line of when the URL reached maximum daily retweets in the Japanese community. This situation is represented in Fig. 5a, with the inverse situation of a Japanese language URL being posted/retweet peak timing amongst English users relative to being posted by a Japanese user at day 0 in Fig. 5b. The peaked shape of the distributions of Fig. 5a at day 0 on the x-axis shows that most English language URLs are posted by Japanese users at similar timing to English users . However, the long tail in the negative day difference (where x< 0) of the x-axis shows that a large proportion of English URLs are posted by Japanese users before English users . Further, the daily retweet peak timing distribution line in orange suggests that these posts are rapidly diffused amongst Japanese users, as many reach their daily retweet peak within the Japanese community shortly after the URL is posted by English users. This is demonstrated by the orange line (retweet daily peak) peaking shortly after the blue line (first posting).

This would suggest that Japanese users do not necessarily find popular English misinformation first before sharing it within their circles. Instead, it is more likely that Japanese users are able to find English misinformation themselves and share it, independently of how popular or obscure that piece of English misinformation is within the English community itself. A similar phenomenon seems to appear for Japanese URLs shared in English, as the trend lines in Fig. 5b follow a similar shape. However, the much lower distribution peak on the y-axis suggests that the volume of URLs is much lower. This was further demonstrated by the sparseness of Fig. 3, showing the low popularity of Japanese URLs amongst English users. We therefore do not consider the volume of Japanese URLs shared by English users to be significant enough to draw the same conclusion.

Our results demonstrate the popularity of English misinformation amongst Japanese users. In addition to being avid spreaders of misinformation in English, they are also highly influential. Most significantly, we show that Japanese users pick up on misinformation in English often before English users themselves. This challenges the notion that Japanese users pick up on popular English misinformation spread by English users. Instead, it suggests that they find their own sources in English to support their agenda independently. This has implications for organizations aiming to combat misinformation in Japanese societies, and posits that they must take a cross-lingual approach in their efforts. Whilst we find minimal evidence of the popularity of misinformation in other languages amongst English users, further work is needed to understand the extent of this phenomena. This includes understanding the popularity of English misinformation in other languages, and non-English misinformation amongst English speakers.

## Discussion

Japan has historically been regarded as having a comparatively low prevalence of misinformation, though hyper-partisan, racist, and deliberately false content still spreads^21^. It is also natural that particular events fuel misinformation, even if they are not partisan or racist in nature, such as in the case of the Great East Japan Earthquake^22,23^. Thus, the nature of the Covid-19 pandemic has forced a shift towards health-based misinformation, which our research points towards. As the founder of Ivermectin is Japanese^5^, this would explain much of the excitement in Japanese Twitter that followed the initial results by ref^6^ in 2020. The popularity of English media amongst the Japanese is also well established^24^, therefore it is of little surprise that much of the misinformation shared by Japanese users regarding Ivermectin came from English sources. What is surprising, as our results showed, is that Japanese users do not rely on English misinformation that is popular amongst English users to disseminate within their circles.

The findings of our top influential users also draw similarities to the aforementioned works that analysed Covid-19 misinformation in other contexts. In the research done by ref^14,15^, a core group of influential users are identified, labelled the “broadcast” group. These are a combination of popular citizen, group, and celebrity accounts. The influential users identified in our results are similar, with the majority of them being popular citizen’s accounts, a few popular group’s accounts, and a small handful of celebrities, well-known reporters, or political commentators. The highly medical nature of the debate surrounding Ivermectin means that many of the popular citizen accounts seen in our results are medical professionals. This result can also be extended to Japanese users, which have their own combination of popular citizen and group accounts within the “broadcast” group.

Our results provide evidence of the popularity of English misinformation amongst the Japanese community, and insight into how it is shared. We also identified the nature of the most influential users responsible for sharing misinformation, finding similarities between English and Japanese users. Understanding how misinformation is shared from different language perspectives is important to making sure that the frameworks and tools that we use to combat misinformation are more inclusive and applicable in a wider range of contexts.

## Methods

### Data collection

**Figure 6:**
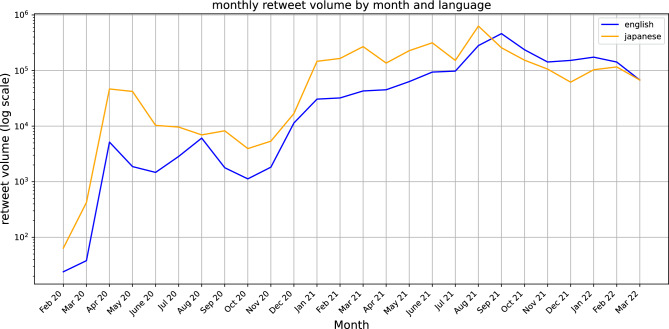
Volume of collected Japanese and English retweets by month.

Publicly available twitter retweets containing the keyword ‘Ivermectin’ in English and equivalent in Japanese from February 2020 to March 2022 were collected using the Twitter REST API v1.1. As this study used publicly available data and no human subjects were involved, it was exempt from ethical review by the Institutional Review Boards of the University of Tokyo and the University of Tsukuba in accordance with the authors’ institutional guidelines. There were a total of 2,094,388 retweets across 698,484 unique users in English, and 3,056,884 retweets across 259,151 users in Japanese. A perfunctory pre-analysis in Fig. 6 shows the initial retweet spike occurring at approximately similar times in Japanese and English in March/April 2020 when the research by ref^6^ was published, and reaching an all-time peak in August/September 2021 when a number of countries around the world were experiencing a high number of cases of Covid-19 before beginning to trail off.

### Content and influential user analysis

A keyword count was performed for each month during the data collection period, with common and expected words such as “ivermectin”, “covid”, and “coronavirus” removed. We made use of the Natural Language Processing spaCy and GiNZA (https://megagonlabs.github.io/ginza/) libraries to perform the same keyword count in Japanese, as such techniques are required to parse content in the Japanese language. To identify influential individual users, node degrees for each user in the dataset were calculated using networkx for each month and language. Users were then ranked by node degree to determine the influentiality of individual users. A higher number of node degrees indicated that the user is retweeted by more unique users, and is thus considered an influential individual.

The similarity of influential users between each month was calculated on the top 10 users with the highest node degrees for each month and language using the Python package rbo (https://github.com/changyaochen/rbo) as an implementation of RBO introduced by Webber et al.^25^. As stated previously, RBO is a rank similarity measure that compares the difference between two lists. It considers top-weightedness by imposing a stronger penalty for differences towards the top of the list, and was initially proposed as a way to compare search query results in search engines. Hence, our RBO calculation is sensitive to changes in highly influential users. This is particularly useful as user influentiality decreases rapidly, therefore changes in highly influential users are an important consideration. The RBO calculation, given two lists of users, *S* and *T*, can be represented as follows:

1 $$\begin{aligned} RBO(S, T, p)=(1 - p)\sum p^{d-1} \cdot A_d \end{aligned}$$

where *p* is a parameter that determines the weight of the top users and *d* determines the depth of the list; both of these are kept constant. $A_d$ is the proportion of overlap between the two lists *S* and *T*. The RBO score varies between 0 and 1, with a score of 0 indicating no similarities in either the presence or rank of users between each month, and a score of 1 meaning that the same users are present at the same rank/level of influence.

Calculating a simple RBO score does not necessarily differentiate between few differences at the top end of the list versus more differences at the bottom of the list. However, the consistency of the RBO calculation between comparison months, and a visual inspection of the tables show that it is a case of the former. RBO also outperforms other common list comparison measures such as Hamming Distance, which would not impose a penalty for differences between users at the top of the list versus users at the bottom. We also do not concern ourselves with the population size difference on the calculation outcome, as the populations are significantly large enough in both languages (698,484 English users vs. 259,151 Japanese users) that the result is still important.

### Cross-lingual analysis

In order to analyse how misinformation was shared between English and Japanese, a multi-step process was used to tag users as “misinformation spreaders”, or pro-use, and thus identify the URLs that misinformation spreaders shared.

**Figure 7:**
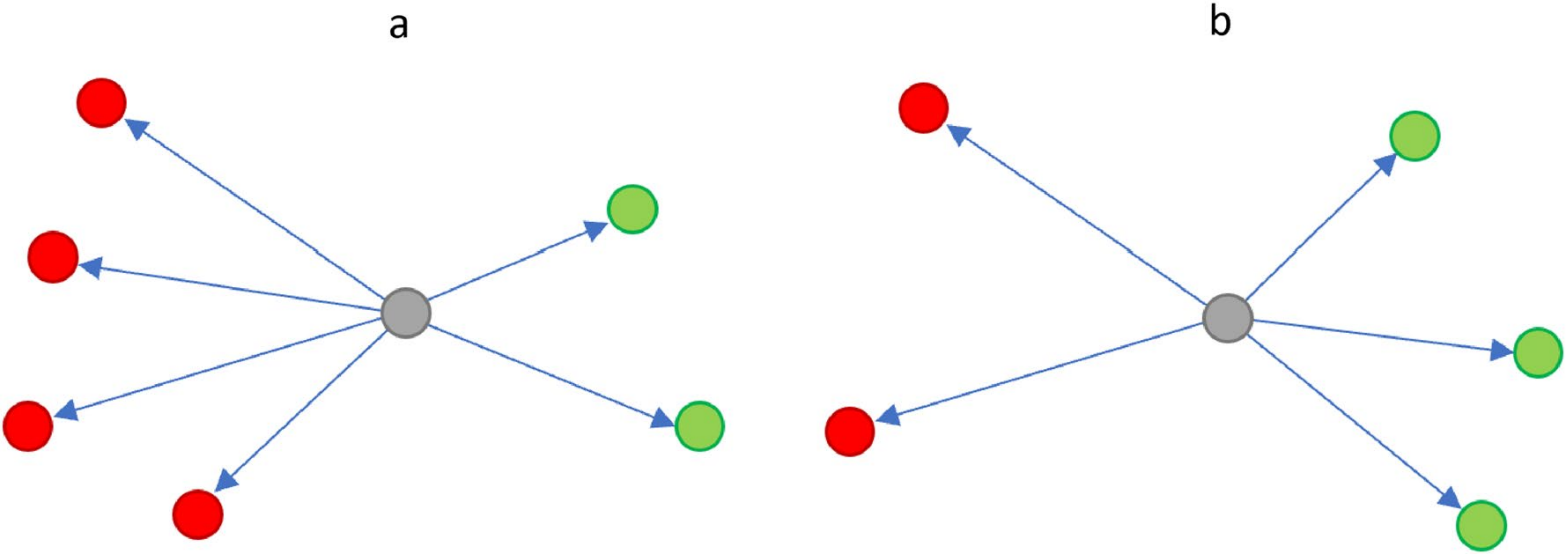
Representation of how users were assigned as pro or anti-use based on interactions with initially tagged influential users. A user (grey) who retweets more pro-use users (red) than anti-use users (green) is considered to be pro-use (scenario a), and vice versa (scenario b).

First, the top ten users for each month from the user influentiality analysis were manually reviewed and tagged as Ivermectin pro-use (“misinformation spreaders”) or anti-use depending on the sentiment expressed in their tweets (Table 6 and 7). We then estimated the stance of remaining users to the next k-hops to get maximum coverage of connected users (Fig. 7). From the starting point of the manually tagged influential users, a given user who retweeted a pro-use user more times than an anti-use user was tagged as pro-use and vice versa. An example of the two scenarios is shown in Fig. 7. The process was repeated to six node hops in English and five node hops in Japanese, achieving a population coverage of 69.7% in English, with 280,713 users tagged as pro-use and 206,428 as anti-use, and 82.6% population coverage in Japanese, with 118,474 users tagged as pro-use and 95,685 as anti-use. The high proportion of users tagged as anti-use despite the proportionally smaller starting point, and the fact that the top links shared by each group support their respective agenda provide a level of confidence in the approach (Tables 8 and 9).

**Table 8:** Top 10 URLs shared by English Ivermectin pro-use users.

Rank	URL	Description	Retweets
1	starpolitical.com/bombshell-report-joe-rogan-says-dr-pierre-kory-treated-200-members-of-congress-with-ivermectin-video/	Bombshell Report: Joe Rogan says Dr Pierre Kory treated 200 members of congress with Ivermectin	7389
2	https://www.nikkei.com/article/DGXZQOFB25AAL0V20C21A1000000/	Translated: Tokyo Medical Association recommends administration of ivermectin	5433
3	https://www.ncbi.nlm.nih.gov/pmc/articles/PMC8383101/	Ivermectin: a multifaceted drug of Nobel prize-honoured distinction with indicated efficacy against a new global scourge, COVID-19	5294
4	https://www.reuters.com/aticle/health-coronavirus-japan-kowa/japans-kowa-says-ivermectin-effective-against-omicron-in-phase-iii-trial-idUSL1N2UB0AV	Japan’s Kowa says Ivermectin effective against omicron in phase III trial	4538
5	htpps://www.oann.com/india-govt-declares-most-populated-state-officially-covid-free-after-widespread-use-of-ivermectin/	India govt declares most populated state officially covid free after widespread use of ivermectin	4445
6	https://www.jpost.com/health-science/israeli-scientist-says-covid-19-could-be-treated-for-under-1day-675612	Israeli scientist says COVID-19 could be treated for under $1\day	4292
7	https://www.emilypostnews.com/p/gofundme-removes-fundraiser-for-dying-b95?r=1im5e&utm_campaign=post&utm_medium=web&utm_source=direct	GoFundMe Removes Fundraiser for Dying Texas Sheriff Deputy After Wife Posts About Ivermectin	4126
8	journals.lww.com/americantherapeutics/ fulltext/2021/08000/ivermectin _for_prevention_and_treatment_of.7.aspx	Ivermectin for Prevention and Treatment of COVID-19 Infection: A Systematic Review, Meta-analysis, and Trial Sequential Analysis to Inform Clinical Guidelines	3930
9	https://www.nobelprize.org/prizes/medicine/2015/press-release/	Press release	3891
10	journals.lww.com/americantherapeutics/ Abstract/9000/Ivermectin_for_Prevention _and_Treatment_of.98040.aspx	Ivermectin for Prevention and Treatment of COVID-19 Infection: A Systematic Review, Meta-analysis, and Trial Sequential Analysis to Inform Clinical Guidelines	3792

**Table 9:** Top 10 URLs shared by Japanese Ivermectin pro-use users, translated to English where original text was in Japanese.

Rank	URL	Description	Retweets
1	https://www.dailyshincho.jp/article/2021/03141057/	Translated: Discoverer of Ivermectin, Dr. Satoshi Omura, appeals for ‘special approval’, Patients taking it in Japan are ‘quickly healed’	16,136
2	https://dailyshincho.jp/article/2021/03211059/	Translated: The reason why pharmaceutical companies stubbornly ‘hide’ the wonder drug ivermectin	14,888
3	https://webronza.asahi.com/science/articles/2021020700003.html	Translated: Ivermectin, discovered by Dr. Ohmura may end the Corona Pandemic	5675
4	https://dailyshincho.jp/article/2021/03201059/	Translated: The Tokyo Medical Association earnestly appeals for the effective drug Ivermectin, which is ‘effective against mutated viruses’	5656
5	https://www.yomiuri.co.jp/choken/kijironko/cknews/20210427-OYT8T50019/	Translated: Whether Ivermectin is effective or not for corona treatment, Japan should take the lead in resolving the global controversy	5265
6	https://kitasato-infection-control.info/	Kitasato University Infection Control Research Centre homepage	5081
7	https://www.nikkei.com/article/DGXZQOFB25AAL0V20C21A1000000	Translated: Tokyo Medical Association recommends administration of ivermectin to prevent worsening symptoms	5031
8	https://anonymous-post.mobi/archives/10115	Translated:<India> Ivermectin leads to a sharp decrease in the number of people infected with the corona virus = WHO “Don’t use ivermectin” = Rapid increase in the number of infected people = 87% decrease in infected people due to repeated use Indian Bar Association “accuses” WHO = Internet reaction “Even if there is no fuss about vaccines, Does that mean that the strongest vaccine made by the Japanese already existed?”	4489
9	https://t.me/Whiplash347/37729	Tokyo’s Medical Assoc. Chairman holds live press conference recommending #ivermectin to all doctors, for all Covid patients.	4398
10	https://dot.asahi.com/dot/2021052600033.html	Translated: Using ‘Ivermectin’ from Japan, India’s corona treatment reduces the number of infected people, but WHO is opposing	4277

Once the users were classified as pro-use or anti-use, URLs shared by pro-use users were then identified. To analyse how misinformation was shared in the cross-lingual context, URLs shared by both English and Japanese pro-use users were extracted, and the language of that URL tagged using the langdetect (https://pypi.org/project/langdetect/) module. We then performed retweet counts for each URL, considering the language of the URL and the popularity through retweet counts by English or Japanese users. We also identified the pro-use users that had retweeted the most unique non-native URLs (i.e. Japanese users retweeting and English language URL), and their influentiality based on the previously calculated node degrees.

Pro-use users were then ordered by the number of URLs shared in their non-native language to produce Fig. 4a. The node degree of the top pro-use users identified in 4a was then plotted in 4b to observe the influentiality of the users that share the most non-native URLs. Due to the high variation in node degrees between each users, we smoothed the results using spline interpolation techniques. The results still show the demonstrably higher average influentiality of Japanese pro-use users.

To analyse the timing of diffusion of misinformation, we compared the timing of when a URL shared by pro-users is first posted in the native language (day 0 in the x-axis of Fig. 5) with when the URL gets posted in the non-native language, and when it reaches peak diffusion (maximum daily retweets) in the non-native language. The purpose of this was to observe how quickly misinformation spreads from one language to another. An asymmetric Laplace distribution was used to fit the retweet timing data, as there are a large number of observations centred around the middle with an exponential decrease as we move away from 0. This indicates that the majority of English URLs are posted by English and Japanese users at around the same time, with long tails on each side due to some URLs that are shared extremely early or late. Sakaki et al. (2012) in their observation of tweet timings in relation to events, specifically earthquakes, fit an exponential Poisson distribution^26^. However, in our case, a significant volume of occurrences happen before the event of a URL being posted by a native user, hence the use of the asymmetric Laplace. Indeed, this volume of occurrences is one of the main contributions of this paper, where we argue that Fig. 5a demonstrates that a significant number of URLs get posted in and reach peak diffusion in Japanese before they are posted by English users for the first time.

A similar diffusion pattern seems to appear for Japanese URLs shared by English users (Fig. 5b); however, manual inspection of these results reveals a number of URLs to YouTube for videos in English. This is likely due to the fact that YouTube uses location information in order to tailor language settings depending on where the site is accessed from. Ignoring YouTube URLs reveals only four Japanese URLs that are posted and reach peak diffusion in English before being posted in Japanese, which is evidently not enough to draw any conclusions about how English users share Japanese URLs. It is therefore likely that there is a a mix of Japanese and English YouTube videos in this analysis. We did not remove them from the main analysis due to the impracticality of verifying the video language of every URL. This was also not a concern for English language URLs due to the significantly larger volume of non-YouTube URLs present.

### Supplementary Information

Supplementary Information 1.

Supplementary Information 2.

## Data Availability

The datasets used in this study are available from the corresponding author on reasonable request. Most can be retrieved from twitter.com, except for deleted tweets. A list of the tweet ids used in this study is also available as supplementary material.
